# Taurine Reverses Oxidative Damages and Restores the Muscle Function in Overuse of Exercised Muscle

**DOI:** 10.3389/fphys.2020.582449

**Published:** 2020-10-26

**Authors:** Anand Thirupathi, Ricardo A. Pinho, Julien S. Baker, Bíró István, Yaodong Gu

**Affiliations:** ^1^Faculty of Sports Science, Ningbo University, Ningbo, China; ^2^Laboratory of Exercise Biochemistry in Health, Graduate Program in Health Sciences, School of Medicine, Pontifícia Universidade Católica do Paraná, Curitiba, Brazil; ^3^Department of Sport, Physical Education and Health, Hong Kong Baptist University, Hong Kong, China; ^4^Faculty of Engineering, University of Szeged, Szeged, Hungary

**Keywords:** taurine, exercise, muscle overuse, oxidative stress, antioxidants

## Abstract

Exercise-induced oxidative stress is linked with the expression level of endogenous antioxidants, but these antioxidants cannot overcome all oxidative stress-related damages in the cells, particularly when cells are under physiological stress. Sometimes, compounds are needed for cellular function, which are produced/activated within the cells, and these compounds can be synthesized by performing exercise, especially high-performance exercise. Taurine is a sulfur-containing amino acid used for various physiological functions. However, its synthesis and accumulation under the oxidative environment may be compromised. Recently, we have shown that taurine level is increased during exercise performance with a decrease in oxidative damage in overused muscles. Other studies have also shown that short-term supplementation with taurine increased physiological performance during severe work intensities, suggesting the role of taurine in improving muscle performance during exercise. However, its precursor cysteine is used in the synthesis of other compounds like GSH and Coenzyme A, which are important for regulating the redox system and energy homeostasis. It is, therefore, important to understand whether taurine synthesis within the cells can blunt the activity of other compounds that are beneficial in preventing oxidative damage during intense exercise. Furthermore, it is important to understand whether taurine supplementation can prevent the conditions observed in the physiological stress of muscles. This review discusses how taurine synthesis could alter exercise-induced ROS generation and the relationship between the physiological stress of muscle and subsequent improvements in exercise performance.

## Introduction

Skeletal muscle is one of the most specialized tissues with plasticity in the human body. It accounts for approximately 40–50% of the total body mass, and it can easily withstand different stimuli from exercise-induced mechanical stress and metabolic stress to other environmental stimuli, including heat exposure and nutrient availability (Rivas and Fielding, 2012; Frontera and Ochala, 2015). These adaptions are linked with various complex systemic interactions such as cellular response, oxidative capacity, and regulation of gene expression and protein level, and this could be achieved through various signaling and structural adaptions (Suhr et al., 2013). Physical exercise is one of the factors that positively promotes these skeletal muscle adaptations. However, strenuous exercise may affect various biochemical and myocellular alterations. For example, high-intensity exercise-induced muscle lesions, following muscle impairment, and muscle adaption. The reasons for muscle overuse injury also include cumulative trauma, which is related to volume and overload intensity or repetitive use and muscle stress.

Reactive oxygen species (ROS) play a crucial role in the exercise-induced pathophysiology of muscle, but it occurs in an exercise-dependent manner (Thirupathi and Pinho, 2018). Moderate exercise is believed to produce a small amount of ROS that is involved in regulating muscular adaption during exercise. However, increased contractile activity of skeletal muscle increases ROS generation which causes various physiological and biochemical adaptions such as mitochondrial biogenesis and changes in active myofibres. ROS is a core factor in regulating both cellular and physiological functions by affecting various signaling or acting as a signaling molecule. Our group has also demonstrated that ROS generation and oxidative stress are involved in trauma-induced muscle injury (Silveira et al., 2013). Further to this, muscle damage and subsequent repair are accompanied by the recruiting of phagocytic cells and macrophages which are additional sources of ROS generation leading to damage of the musculature itself and muscle impairment.

Muscle-induced oxidative damage is often unavoidable and requires therapeutic interventions, e.g., supplementary foods containing antioxidants, that regulate the redox process (Waters et al., 2010; Molnár et al., 2016). However, some of these supplements may reduce the beneficial effects of exogenous antioxidants during strenuous exercise, and selecting a proper supplementary nutrient is necessary to maintain muscle performance (Harty et al., 2019). Taurine is one such supplement that enhances cellular functions in several ways, e.g., stabilizing the membranes and regulating the cell volume, balancing redox homeostasis, and controlling ion channels (Schaffer et al., 2014; De Luca et al., 2015; Lambert et al., 2015). In addition, taurine plays a significant role in reducing the oxidative stress and inflammatory responses induced by exercise. Taurine is a sulfur-containing free amino acid present in the body with concentration ranges between 5 and 20 μm/g, particularly in excitable tissues such as muscles, but it can also be obtained from external sources like meat and sea-food (Huxtable, 1992, 2000; Schaffer et al., 2010). Studies have shown that overuse of muscle depletes taurine level and the loss of taurine occurs mostly in the fast-twitch fibers (Dawson et al., 2000; Matsuzaki et al., 2002; Yatabe et al., 2003). Therefore, taurine supplements are necessary to compensate for intracellular taurine levels and to increase exercise performance. However, the duration of taurine administration under chronic exercise conditions is yet to be established.

### Muscle Overuse and Synthesis of Taurine Under ROS Environment

**FIGURE 1:**
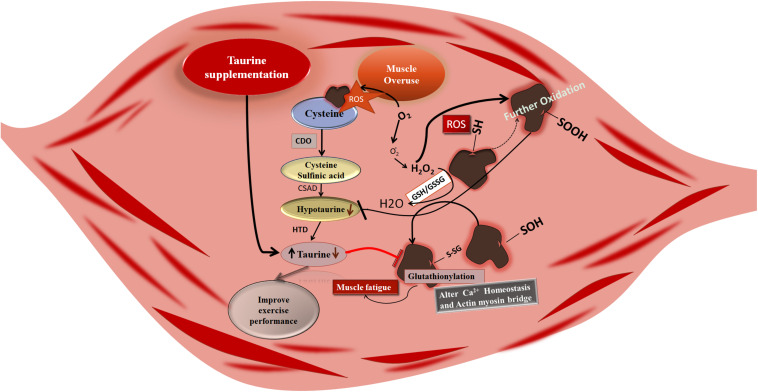
Muscle overuse-induced cysteine oxidation and subsequent intermediary compound sulfinic acid oxidation compromising the synthesis of taurine and glutathione (GSH). Decreased levels of taurine and GSH may inhibit the glutathionylation resulting in reversible oxidation of cysteine. Further, disturbances in the glutathionylation may cause disturbances in the muscle function like Ca^2+^dyshomeostasis and actin myosin bridge alteration which results in muscle fatigue; taurine supplementation increases the level of taurine in the muscle resulting in regulating Ca^2+^homeostasis and actin myosin bridge activation by controlling ROS and improving exercise performance. CDO, cysteine dioxygenase; CSAD, cysteine sulfinic acid decarboxylase; HTD, hypotaurine dehydrogenase.

Muscle overuse affects taurine synthesis by producing ROS, and consequently, taurine-induced benefits are compromised. Taurine biosynthesis is achieved as a result of the following biochemical reactions: oxidation of cysteine by cysteine dioxygenase to form cysteine sulfinic acid; followed by decarboxylation of cysteine sulfinic acid by the cysteine sulfinic acid decarboxylase producing hypotaurine, and finally the oxidation of hypotaurine to taurine. Exercise-induced ROS generation can alter this sequence (Huxtable, 1992; Faggiano et al., 2005). As cysteine is a less abundant amino acid, its utilization for protein and non-protein synthesis can compromise taurine synthesis. However, exercise-induced ROS generation may primarily attack the sulfhydryl (-SH) terminal of cysteine, which is the location where several cellular events are organized for the synthesis of proteins including taurine (Kolossov et al., 2015), and thus exercise-induced ROS is an important factor for organizing the above-mentioned scenario (Figure 1). For example, muscle overuse-induced cysteine oxidation and its intermediate cysteine sulfinic acid oxidation result in a reduced level of taurine and glutathione synthesis (GSH), ultimately compromising the taurine-induced benefits in the muscle, such as influencing GSH level and glutathionylation (Bertolone et al., 2020). Glutathionylation caused inhibition of reversible cysteine oxidation is compromised, which may fail to protect from oxidative damage during muscle contraction (Qin et al., 2013). However, the level of ROS is regulated by exercise-induced adaptation in the cells which may prevent further ROS-induced damage (Scheele et al., 2009; Kawamura and Muraoka, 2018).

Exercise affects the taurine turnover (Dawson et al., 2002; Matsuzaki et al., 2002), but it also varies between person to person due to the amount of protein intake or protein synthesis. Exercise-induced ROS generation also influences the protein synthesis-related signaling (Vaanholt et al., 2008). Furthermore, several other intermediate compounds, such as folic acid and enzymes like methyl tetrahydrofolate reductase (MTHFR), influence the availability of cysteine and further taurine synthesis, which are all affected by exercise-induced ROS generation (De Luca et al., 2015). Since glutathione and taurine share the same precursor, taurine synthesis may affect GSH synthesis which can increase the oxidative damages induced by overuse of muscles. Retaining taurine concentrations at the cellular level is important for obtaining exercise-induced benefits and in preventing exercise-induced muscle damage.

### Does Taurine Regulate Mitochondrial ROS Production in Overused Muscle?

Exercise endurance is proportional to the mitochondrial function (Hood et al., 2019) and the mitochondrial membrane potential is partially responsible for mitochondrial function, regulating the intracellular ionic charges (Thirupathi et al., 2018). Hansen et al. have shown that taurine supplements can regulate proper proton pumping without affecting mitochondrial matrix alkalinity (Hansen et al., 2010). We have also shown that taurine supplementation maintained mitochondrial membrane potential in the overuse of muscle (Thirupathi et al., 2018). Muscle overuse-induced mitochondrial ROS is also prevented by taurine supplementation. For example, ROS is produced in several ways in the mitochondria, and succinate oxidation is one of the ways to generate ROS during NAD-linked substrates, such as coupled respiration with succinate in complex I via reverse electron transfer from complex II to I.

**FIGURE 2:**
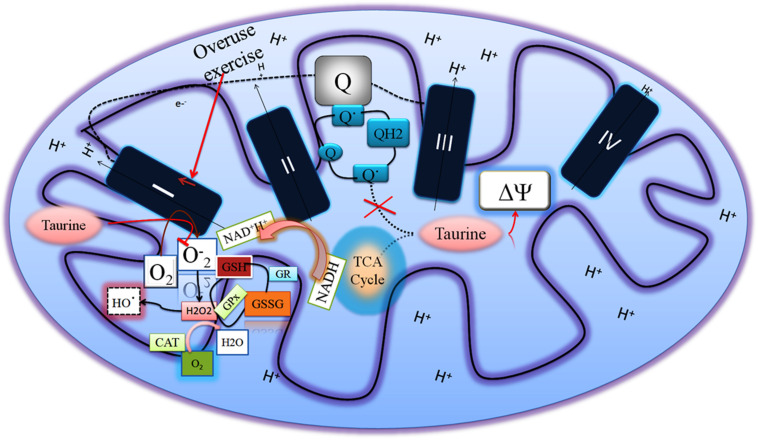
Overused muscles increase the complex I activity to compensate for energy deprivation, which increases superoxide radical generation. Taurine regulates the mitochondrial membrane potential and decreases both the superoxide radical in the complex I and semiubiquinone radical so as to increase the muscle performance.

Our group has shown that taurine supplementation reduced ROS in overuse of muscles exposed to succinate, which produced mtH_2_O_2_ through regulating mitochondrial membrane potential and preventing the formation of semiubiquinone radical (Thirupathi et al., 2018). However, taurine does not increase the activity of other complexes except complex I in the ETC in overuse muscle, suggesting that taurine prevents ROS production at the complex I level (Figure 2). Overused muscles demand energy levels that are as high as possible at the initial level of onset of exercise to overcome strenuous exercise-induced energy deprivation. This results in increased complex I activity and ultimately produces ROS due to possible electron flow blockage and reduction in the ubiquinone pool. This limits ATP production, and consequently reduces the performance of exercise and causes other dysfunctions in the muscles (Brand, 2016). Taurine can change this scenario at the complex I level to improve muscle function and exercise performance.

### Does Taurine Prevent DNA Damage in Overused Muscle?

Muscle overuse releases a higher level of ROS and alters inflammatory cascades which ultimately leads to DNA damage and reduces DNA repair protein expressions. This can be prevented by long-term exercise by obtaining cellular adaptation (Neubauer et al., 2008; Soares et al., 2015). Although studies have shown the effect of taurine on regulating inflammatory processes and ROS generation (Lin et al., 2013; Galan et al., 2018), taurine’s role in reducing DNA damage is poorly understood particularly in the exercise overuse situation. We have shown that taurine administration at 150 mg/kg body weight decreased the DNA damage in overused muscles (Thirupathi et al., 2018). Taurine prevented the exercise-induced nitrosative inflammation and DNA damage through the NF-kB signaling pathway (Sugiura et al., 2013). Drug-induced taurine deficiency may alter several signaling activation such as the PI 3-kinase/AKT pathway that is linked with DNA damage-induced cell death (Pastukh et al., 2005). Hypoxic state-induced DNA damage is a common phenomenon, especially during muscle overuse. This may be linked with a low level of Ca^2+^ (Zhang et al., 2004; Husain and Mahmood, 2020). Taurine’s role against hypoxic injury is achieved through regulating Ca^2+^homeostasis (Schaffer et al., 2002). Therefore, taurine could be a potential candidate for preventing ROS-induced DNA damage in the overused muscle.

### The Role of Taurine in the Muscle Phenotype Under Exercise Conditions

Taurine increases the muscle level, and this could be due to phenotypic-specific contractile properties (De Luca et al., 2015; Terrill et al., 2016). Supplementation of taurine with a higher content of amino acid in the skeletal muscle leads to an increase in muscle chloride channel conductance (gCl). Taurine transporter (TauT) expression during myogenesis is increased, and taurine stimulates the myofibre differentiation (Uozumi et al., 2006; Miyazaki et al., 2013). Although these studies showed the importance of taurine in muscle phenotype, the mechanism that regulates the muscle phenotype is unclear. The possible mechanism that regulates the muscle phenotype is mitochondrial biogenesis and its role in tissue development (De Luca et al., 2015). Taurine could protect the susceptibility of exercise-induced muscle damage by controlling oxidative stress and inflammation. Stress-induced muscle damage and subsequent taurine treatment increased the susceptibility to muscle damage by up to 40% or more (Terrill et al., 2016; Ra et al., 2016; Waldron et al., 2019). However, taurine could not protect the muscle damage in the initial stretching, and it can protect the subsequent pathology, which is associated with initial injury through regulating inflammatory response and oxidative stress. Additional studies are required in order to find a taurine induced specific mechanism that supports to protect exercise-induced muscle damage and necrosis.

The overuse of muscle alters muscle proliferation and regeneration capacity, and several muscle-specific proteins are involved in regulating the muscle phenotype, including MyoD, Myf5, and myogenin (Flann et al., 2011; Baumert et al., 2016). Exercise is believed to activate these muscle-specific proteins to regulate the muscle phenotype, however, how these proteins are organized to reverse the overuse injury in muscle into normal is ambiguous. Muscle overuse-induced ROS could be the main contributory factor that alters the muscle regulatory protein expression, compromising the muscle function. Taurine can alter these scenarios by regulating calcium uptake and inflammatory mediators and maintaining optimal ROS generation (Miyazaki et al., 2013; Nam et al., 2017). Increased ROS generation facilitates various adaptation in the muscle cells for increasing force generation, and its concentration yet to be determined and is one of the limitations of the ROS- induced benefits (Radák et al., 2001; Thirupathi et al., 2018, 2020). We recently demonstrated that taurine-associated gold nanoparticle increased the Myf-5 protein in the overuse of the muscle to protect the muscles from further damage, suggesting that taurine associated gold nanoparticle may optimally maintain the ROS level so as to increase the ROS-induced benefits, such as regulating muscle regulatory proteins (De Luca et al., 2003; Warskulat et al., 2004; Goodman et al., 2009; Silva et al., 2011; da Silva et al., 2014; Horvath et al., 2016; Terrill et al., 2016). Furthermore, the induction of the Myf5 protein requires amino acid methylation, particularly arginine and cysteine, and ROS could mediate cysteine methylation, which ultimately affects the synthesis of taurine. How these mechanisms are orchestrated under conditions of muscle overuse is still unknown.

### Taurine’s Role in Exercise Performance

Taurine regulates several physiological functions. Since its discovery in ox bile in 1827, understanding of taurine’s role in increasing exercise performance is limited. Studies have shown that the administration of taurine has a significant effect on exercise performance, but other studies have demonstrated that taurine did not influence exercise performance. For example, 1 g of taurine administration improved the 3-km time trail running before the exercise, whereas during the 4-km running trail did not have a significant effect on exercise performance with taurine supplementation (Balshaw et al., 2013; Ward et al., 2016). This may be due to the exhausting performance increasing ROS generation at a threshold activity, and further work on the effects of taurine and the duration and intensity of exercise need to be established. Therefore, a decrease in muscle contraction and relaxation and experimental protocol using correct dosages of taurine may optimally maintain the ROS generation in the muscle cells, which ultimately improves exercise performance. Compounds such as GSH and coenzyme A may reduce the benefits of taurine, as they are utilized to synthesis other compounds. Keeping optimal taurine levels in the muscle cell is difficult, and facilitating taurine synthesis in the cells may advance the role of ROS-induced benefits through taurine synthesis instead of ROS-induced muscle damage (De Luca et al., 2003; Ito et al., 2010; McLeay et al., 2017).

The effect of the duration of taurine administration in increasing exercise performance is ambiguous. For example, oral administration of taurine may promote the exercise performance initially, but long-term exercise using taurine does not have a significant effect, suggesting that oral intake of taurine at the initial level may promote the exercise performance, but longer duration of exercise may not retain the cellular taurine levels (Rahman et al., 2011; De Carvalho et al., 2017; Sajid et al., 2017; Niu et al., 2018; Li et al., 2019; Seidel et al., 2019). However, strenuous exercise-induced muscle damage may be reversed and the duration of taurine administration and effects on chronic exercise conditions need to be established to evaluate the total beneficial effects of taurine supplementation.

Exercise training in terms of type, duration, and intensity perturbs the oxidative stress status and amino acid pool, including taurine in athletes. This demands additional supplements to improve exercise performance. Studies have shown that taurine supplementation improved the exercise performance, and the mechanism that is involved to improve the muscle performance is following: (I) increased concentrations of taurine in the plasma increase the calcium uptake and release to contractile filaments, which further enhances the force production; (II) regulating muscle membrane; and (III) increasing the mitochondrial buffering (El Idrissi, 2008; Galloway et al., 2008; Batitucci et al., 2018). Speed and intensity during exercise may be achieved through an increased level of taurine, which may indicate its release into muscle fibres (Ward et al., 1999). The nature of the increase in taurine concentration in the plasma content after strenuous exercise must be established, as this may be the indication of muscle damage, muscle fatigue, or osmolarity adaptation within the blood (Ward et al., 1999). The release of taurine from the muscle and other blood constituents may disturb the level of taurine. For example, changes in the plasma osmolarity due to exercise can affect the distribution of taurine in the plasma, and plasma volume decreases post-marathon. Taurine and its corelease of water maintain plasma volume and may redistribute the taurine level to improve performance (Ward et al., 1999).

### Metabolic Action of Taurine in the Skeletal Muscle

Taurine’s metabolic action in maintaining normal physiological homeostasis may be related to its action on glucose uptake, insulin sensitivity, and further fat oxidation in the skeletal muscle. Studies have shown that taurine improved insulin sensitivity in the fructose-fed rat to stimulate glycolysis and gluconeogenesis (Mozaffari et al., 1986). Taurine deficiency showed an increased level of glucose uptake during treadmill running and glycolysis, resulting in elevated lactate production, which further contributed to impairing the exercise performance (Ito et al., 2014). As an energy sensor molecule, AMPK plays a crucial role in fatty acid oxidation and the peroxisome proliferator-activated receptor α (PPARα), which regulates the oxidation and transport of fatty acids. AMPK-activated subunits and the level of PPARα are reduced in the TauTKO muscle, suggesting that taurine is crucial for regulating energy metabolism during exercise in the skeletal muscle (Ito et al., 2014). Taurine supplementation may restore the energy homeostasis under pathological situations, whereas it improves muscle performance under normal conditions. For example, in one study, dietary supplementation with taurine attenuated the oxidative stress and inflammatory responses in patients with type 2 diabetes mellitus (T2DM) (Maleki et al., 2020).

### Effect of Taurine on Antioxidant Enzymes in the Skeletal Muscle

Taurine affects the availability of antioxidant enzymes such as superoxide dismutase (SOD), catalase (CAT), and glutathione peroxidase (GPx), but its effect on antioxidant enzymes depends on its concentration. Taurine has increased the antioxidant enzyme activity in a dose-dependent manner in malathion-induced oxidative stress (Ince et al., 2017). It also formed taurine chloramine (TauCl), which may influence the availability of antioxidant enzymes in a dose-dependent manner. TauCl inhibits the overproduction of superoxide radicals in a dose-dependent manner (Kim and Cha, 2009). Studies have shown that TauCl activates the Kelch−like ECH−associated protein 1–nuclear factor E2−related factor 1 (Keap1–Nrf2) pathway (Sun Jang et al., 2009; Kim et al., 2010) and Nrf2 target genes including SOD, CAT, and heme oxygenase-1 are activated. The taurine independently affects the Nrf1-ARE pathway affecting the antioxidant enzymes such as heme oxygenase-1. However, taurine supplementation does not influence the antioxidant enzyme level in the skeletal muscle following eccentric exercise. It can promote the synthesis of GSH, which results to increase the action of GPx, and this could be the mechanism of antioxidant defense (Silva et al., 2011; da Silva et al., 2014).

## Conclusion

Muscle overuse may require a patient to take additional supplements, the function of which is to alter the inflammatory response and reduce oxidative stress without affecting muscle function. However, the duration of supplement administration must be established and monitored. Taurine reverses muscle function under overuse conditions in several ways, including by controlling mitochondrial ROS production, regulating membrane potential, preventing DNA damage, and inducing muscle regulatory proteins. ROS could be the mediator in organizing these conditions. Synthesis of taurine as a means of endogenously improving exercise performance is a major task. Optimal ROS generation due to exercise may facilitate the synthesis of taurine in various ways, such as by primarily attacking sulfhydryl (-SH) terminal of cysteine, the location where several cellular events are organized including the synthesis of taurine. However, how this is achieved through exercise-mediated ROS is yet to be established. Furthermore, the mechanism that drives ROS-induced effects in combination with taurine supplementation must be established in both human and animal models.

## Author Contributions

AT, RP, JB, BI, and YG conceived the idea and wrote the manuscript. All the authors contributed to the final version of the manuscript.

## Conflict of Interest

The authors declare that the research was conducted in the absence of any commercial or financial relationships that could be construed as a potential conflict of interest.
